# Metabolism-regulating non-coding RNAs in breast cancer: roles, mechanisms and clinical applications

**DOI:** 10.1186/s12929-024-01013-w

**Published:** 2024-02-26

**Authors:** Shiliang Xu, Lingxia Wang, Yuexin Zhao, Tong Mo, Bo Wang, Jun Lin, Huan Yang

**Affiliations:** 1 Department of Clinical Laboratory, The Second Affiliated Hospital of Soochow University, Suzhou, Jiangsu 215004 People’s Republic of China; 2 Department of Oncology, The Second Affiliated Hospital of Soochow University, 1055 Sanxiang Road, Suzhou, Jiangsu 215004 People’s Republic of China

**Keywords:** Non-coding RNAs, Metabolism, Glycolysis, Lipid metabolism, Amino acid metabolism, Breast cancer

## Abstract

Breast cancer is one of the most common malignancies that pose a serious threat to women's health. Reprogramming of energy metabolism is a major feature of the malignant transformation of breast cancer. Compared to normal cells, tumor cells reprogram metabolic processes more efficiently, converting nutrient supplies into glucose, amino acid and lipid required for malignant proliferation and progression. Non-coding RNAs(ncRNAs) are a class of functional RNA molecules that are not translated into proteins but regulate the expression of target genes. NcRNAs have been demonstrated to be involved in various aspects of energy metabolism, including glycolysis, glutaminolysis, and fatty acid synthesis. This review focuses on the metabolic regulatory mechanisms and clinical applications of metabolism-regulating ncRNAs involved in breast cancer. We summarize the vital roles played by metabolism-regulating ncRNAs for endocrine therapy, targeted therapy, chemotherapy, immunotherapy, and radiotherapy resistance in breast cancer, as well as their potential as therapeutic targets and biomarkers. Difficulties and perspectives of current targeted metabolism and non-coding RNA therapeutic strategies are discussed.

## Introduction

Breast cancer, the most prevalent cancer in women and a leading cause of cancer-related deaths, has surpassed lung cancer in prevalence among women according to 2020 Global Cancer Statistics [1]. Treatment decisions are significantly influenced by molecular typing and histologic features. Molecular classifications include luminal A, luminal B, HER2-enriched, and triple-negative breast cancer. Histologically, invasive ductal carcinoma is the most common, followed by invasive lobular carcinoma. These classifications impact prognosis and treatment options [2]. Breast cancer management involves local and systemic therapies. Local treatment includes surgical removal and radiotherapy. Systemic therapy varies based on subtypes such as endocrine therapy, HER2-targeted therapy, chemotherapy, and immunotherapy.

Tumors exhibit a distinct metabolic reprogramming, a hallmark characterized by the Warburg effect [3–5]. In the dynamic tumor microenvironment, cells adjust their metabolism to efficiently use glucose, lipids, and amino acids for rapid proliferation, survival, and metastasis. Despite lower ATP efficiency, the Warburg effect in cancer cells supports their high energy demands [6]. Increased glycolysis directs intermediates to biosynthetic pathways, promoting the synthesis of lipids, amino acids, and nucleosides for cell growth [7]. Tumor cells also display a 'lipogenic phenotype', enhancing fatty acid synthesis independently of exogenous sources [8]. While aerobic glycolysis predominates, some carbon is redirected to the tricarboxylic acid (TCA) cycle via glutamine metabolism, contributing to energy cycling and fatty acid synthesis [6, 9]. The intertwined reprogramming of these pathways collaborates to facilitate tumor growth and proliferation.

Non-coding RNAs (ncRNAs) are functional transcripts without protein-coding potential [10, 11]. They play key roles in developmental and pathological processes involving chromatin remodeling, transcription, post-transcriptional modifications and signal transduction [12]. Breast cancer exhibits a significant number of differentially expressed ncRNAs, some of which are linked to specific subtypes [13–16]. For instance, the differential expression of the *miR-99a/let-7c/miR-125b* miRNA cluster distinguishes between Luminal A and B subtypes [13]. NcRNAs participate in metabolic reprogramming by regulating nutrient transport and utilization, influencing glucose, fatty acid, and amino acid metabolism [17]. Metabolic disorders mediated by ncRNAs ultimately manifest clinically as treatment resistance with poor prognosis. Here, we review the roles and mechanisms by which ncRNAs regulate metabolic reprogramming in breast cancer. Additionally, we discuss the impact of metabolism-regulating ncRNAs on the sensitivity of existing therapies and their potential application as new therapeutic targets and biomarkers.

## Characterization, biogenesis and biology of ncRNAs

**Fig. 1:**
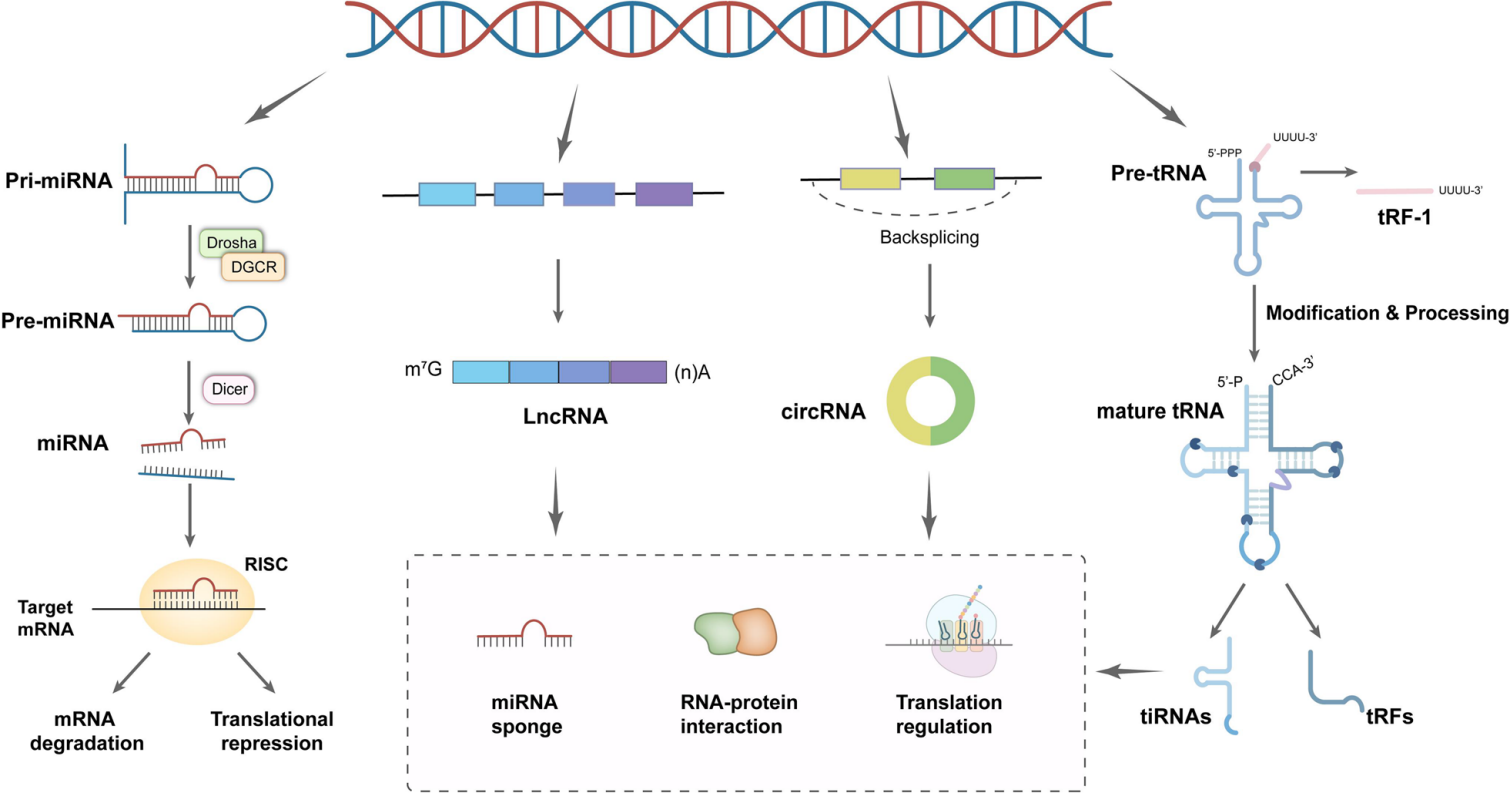
Characterization, biogenesis and biology of ncRNAs. Biogenesis of miRNAs, LncRNAs, circRNAs and tsRNAs and the molecular mechanisms of their biological functions

About 75% of the human genome is transcribed into RNA, while only 3% is transcribed into protein-coding mRNA [18]. RNAs that do not code for proteins, known as non-coding RNAs, can be divided into different categories based on length, shape, and source. Among these categories, microRNA (miRNA), long non-coding RNA (LncRNA), circular RNA (circRNA), and tRNA-derived small RNA (tsRNA) are the four main types of non-coding RNAs, each with different functions in breast cancer (Fig. 1).

MiRNAs are endogenously produced 21–23 nucleotide-long non-coding RNA molecules [19]. The most original forms of miRNAs are primary miRNAs (pri-miRNAs). Pri-miRNAs are precisely cleaved by the Drosha/DGCR complex into precursor miRNAs (pre-miRNAs) with a stem-loop structure, which are then transported into the cytoplasm. Once in the cytoplasm, the Dicer enzyme cleaves the pre-miRNA, releasing the mature and biologically active miRNA strand [20, 21]. In a typical functional mechanism, the mature miRNA strand binds to argonaute (Ago) to construct miRNA-induced silencing complex (miRISC). The miRNA directs miRISC to partially pair-bind on the 3'UTR of the target mRNA, resulting in degradation or translational repression of the complementary mRNA [22, 23].

LncRNAs are single-stranded and longer than 200 nucleotides [24]. LncRNAs are primarily transcribed by RNA polymerase II (Pol II) or other RNA polymerases. They are often capped by 7-methyl guanosine (m7G) at their 5′ends, polyadenylated at their 3′ends, and spliced similarly to mRNAs [25]. After genomic DNA is transcribed to generate LncRNAs, some function in the nucleus, while others are translocated to the cytoplasm, where they can regulate mRNA stability, translational efficiency, and interfere with post-translational modifications of proteins [26].

CircRNAs are also more than 200 nucleotides in length, exist as closed-loop RNAs, and lack polyadenylated tails compared to mRNAs [27]. CircRNAs are produced by non-canonical splicing events called reverse splicing. During reverse splicing, a downstream splice-donor site is covalently linked to an upstream splice acceptor site. circRNAs are highly stable due to their covalent closed-loop structure, which protects against exonuclease-mediated degradation [28]. They can act as miRNA sponges or competitive endogenous RNAs (ceRNAs), competing with other RNAs for miRNA pairing. CircRNAs can also regulate transcription in the nucleus and bind to protein factors [29].

TsRNAs, small non-coding RNAs of approximately 13–48 nucleotides in length, are derived from the precise processing of mature tRNA or pre-tRNA. The biogenesis of tsRNA can occur at all stages of tRNA maturation [30–32]. TsRNA consists of two main components: tRNA halves and tRNA-related small RNA fragments (tRFs). TRNA halves are often known as tRNA-derived stress-induced RNAs (tiRNAs) because their biogenesis primarily occurs under stress conditions such as hypoxia, oxidative stress, heat shock, and nutrient deprivation. Mature tRNA is cleaved into 5′-tRNA halves (5′-tiRNA) and 3′-tRNA halves (3′-tiRNA) by angiogenin (ANG) at the anticodon loop [33–35]. tRF includes tRF-5, tRF-3, tRF-1 and tRF-2. tRF-5, tRF-3 and tRF-2 are products of the cleavage of mature tRNAs, whereas tRF-1 is cleaved from pre-tRNAs. tRF-2s are a newly discovered type of tRFs, generated from the internal regions of mature tRNA spanning variable-length anticodon regions [36, 37]. The current understanding of the biological functions of tsRNAs is categorized into three main groups: RNA silencing, translational regulation, and epigenetic regulation. Firstly, tsRNAs can affect RNA splicing by targeting the 3′-UTR region of mRNA or by targeting mRNA for competitive binding. Next, YB-1 binding tRFs inhibit global translation by displacing translation eukaryotic initiation factors and inducing the assembly of stress granules. tRFs can also regulate translation by interacting with ribosomes. In addition to this, tRFs can inhibit LTR-retrotransposons or participate in non-coding RNA regulation [38, 39].

There is growing evidence that aberrant expression of ncRNAs is implicated in the initiation and progression of breast cancer. These ncRNAs have high stability in vivo and represent potential biomarkers for diagnosis and prognosis. During tumorigenesis, ncRNAs actively contribute to the metabolic reprogramming of tumors. They play a role in modulating key metabolic pathways, such as glucose metabolism, lipid metabolism and amino acid metabolism, by interacting with proteins or regulating the expression of metabolism-related genes. Therefore, an in-depth study of the relationship between ncRNAs and metabolic reprogramming could provide important clues for the development of new therapeutic strategies and biomarkers for breast cancer. Targeted therapies and metabolic interventions for the metabolic characteristics of breast cancer cells may become an important direction for the treatment of breast cancer in the future.

## Non-coding RNA and breast cancer glucose metabolism

**Fig. 2:**
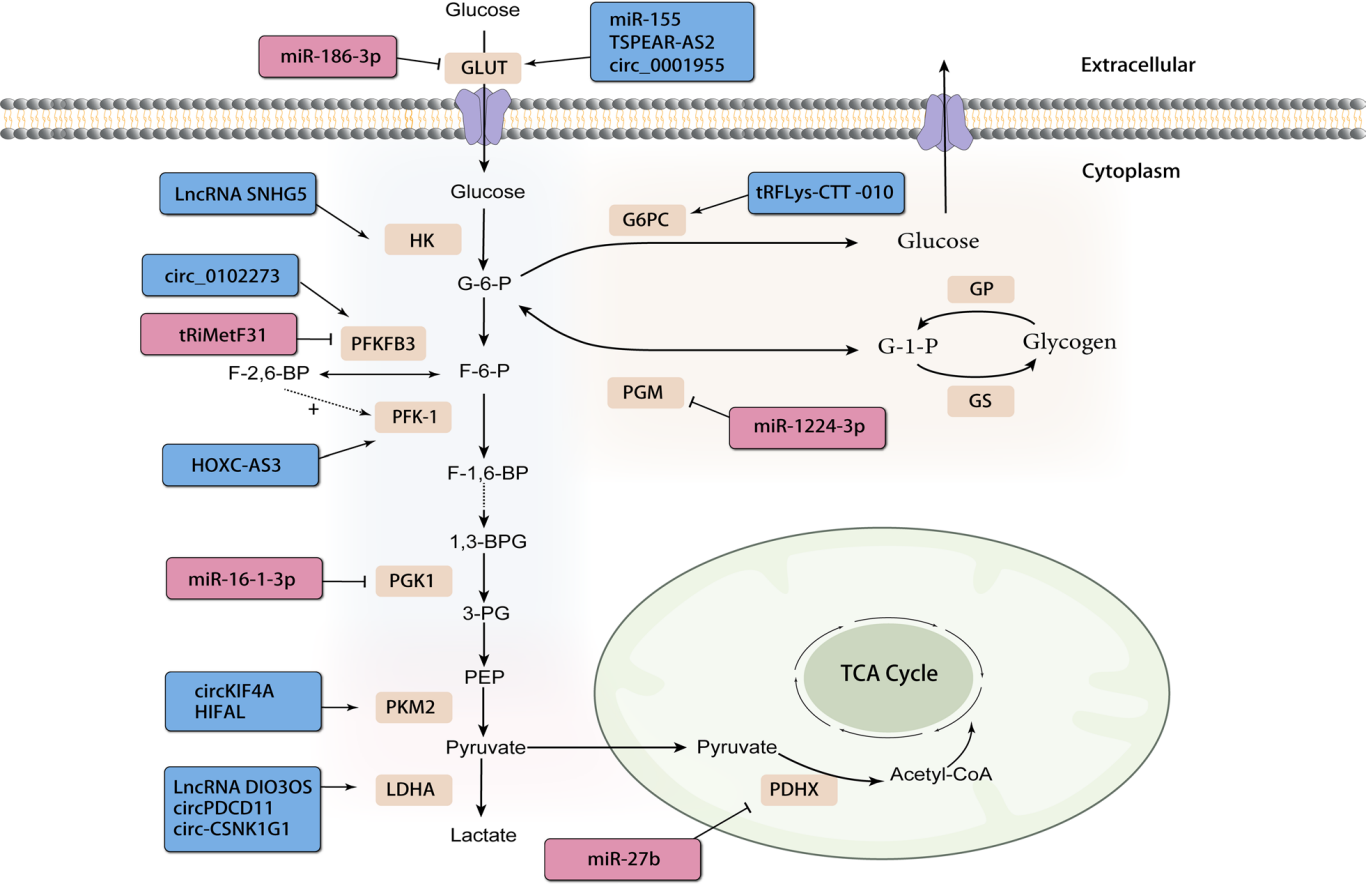
Steps in ncRNA-regulated reprogramming of glucose metabolism in breast cancer by glycolysis, lactate formation, glycogen synthesis and catabolism and the tricarboxylic acid cycle. Blue boxes and arrows represent positive regulations, while red boxes and lines with blunt ends represent negative regulations

Non-coding RNAs will impact various stages of glucose metabolism, including glucose transport, the glycolytic pathway, glycogen synthesis and catabolism in the cytoplasm, and the tricarboxylic acid cycle in the mitochondria, as detailed in the following sections (Fig. 2; Table 1).

### Glucose transporters

Solute carriers (SLCs) are integral membrane transport proteins responsible for transporting nutrients, ions, or other metabolites across cell or organelle membranes. In the SLC superfamily, glucose transporter proteins facilitate the entry of glucose across hydrophobic cell membranes. These solute carriers can be categorized into two families: the secondary active Na+/glucose cotransport proteins (SGLT, gene family solute carrier *SLC5A*) and the facilitative glucose transporter proteins (GLUT, gene family *SLC2A*) [40]. Numerous studies have demonstrated that glucose transporter proteins GLUT1 and GLUT3 play a pivotal role in increasing glucose uptake in tumor cells, leading to a high rate of glucose conversion to lactate [41, 42]. For instance, Kim et al. discovered that *miR-155*-deficient breast cancer cells exhibit reduced mitochondrial oxygen consumption and impaired proliferation under low glucose conditions. Through targeted metabolomics and quantification of glycolysis-related markers, they found that *miR-155*-deficient cells displayed low expression of GLUT1 and GLUT3, suggesting that miR-155 can influence glucose metabolism by altering the gene expression of glucose transporters [43]. In another study, *LncRNA TSPEAR-AS2* was found to bind to *GLUT1* mRNA and enhance its stability. Knockdown of *TSPEAR-AS2* significantly reduced glucose uptake, lactogenesis and ATP production in BC cells, leading to a decrease in extracellular acidification rate (ECAR), a measure of total intracellular glycolytic flux. Further experiments confirmed that *TSPEAR-AS2* mediated aerobic glycolysis by regulating the IGF2BP2/GLUT1 axis [44]. Hong Chen et al. observed that the down-regulation of *circ_0001955* significantly inhibited glucose consumption, lactate production, and ATP levels in BC cells. *Circ_0001955* has a specific binding site with *miR-1299*, which can bind to the *GLUT1* 3'UTR. It was ultimately confirmed that *circ_0001955* regulates the *miR-1299/GLUT1* pathway, leading to tumor-suppressing effects such as decreased cell proliferation, angiogenesis, migration, invasion, and glycolysis [45]. Similarly, non-coding RNAs like *circ_0072995, circ-TFF1*, and *LINC00346* in breast cancer cells enhance the efficiency of glucose uptake and glycolysis by upregulating the protein levels of GLUT1 [46–48].

In addition to affecting the function of GLUT1, non-coding RNAs also influence GLUT3. A recent study revealed that epidermal regulator (EREG) acts as an agonist of EGFR, activating EGFR signaling and several downstream glycolytic genes, including glucose transporter protein type 3 (*GLUT3*), hexokinase 2 (*HK2*), and pyruvate dehydrogenase kinase 1 (*PDK1*) [49, 50]. He and his colleagues wondered whether EREG expression in breast cancer cells was post-transcriptionally regulated by any miRNA. They eventually discovered that *miR-186-3p* directly targets EREG and downregulates the glycolytic gene *GLUT3*, thereby inhibiting aerobic glycolysis [51].

### Major pathways of glucose metabolism

#### Glycolysis

Glycolysis serves as the common initiation pathway for both anaerobic and aerobic oxidation of glucose [52]. Within glycolysis, there are three irreversible reactions catalyzed by hexokinase (HK), phosphofructokinase-1 (PFK-1), and pyruvate kinase (PK), respectively. These reactions have the slowest rates and are controlled by the three key enzymes regulating the flow of glycolysis (Fig. 2).

HK is the first key enzyme in the glycolytic pathway. Once glucose is transported into the cell by transporter proteins, it can be rapidly phosphorylated by HK, forming glucose-6-phosphate (G6P). G6P becomes trapped within the cell and plays a role in downstream metabolic pathways [53]. A study demonstrated that BACH1 binds to the promoters of glycolytic genes, such as *HK2* and glyceraldehyde-3-phosphate dehydrogenase (*GAPDH*), activating their expression and promoting the rate of glycolysis, thus facilitating tumor cell metastasis [54]. Huang et al. found that *LncRNA SNHG5* upregulated BACH1 expression in breast cancer cells by sponging on *miR-299*. Silencing *SNHG5* led to reduced protein expression of HK2, PFK1, and GAPDH, resulting in a significant decrease in glucose consumption and lactate production [55].

PFK-1 serves as the 'gatekeeper' of glycolysis by catalyzing the conversion of fructose 6-phosphate (F6P) into fructose 1,6-bisphosphate(F-1,6-BP) [56]. *LncRNA HOXC-AS3* was an oncogenic lncRNA associated with poor prognosis in breast cancer that could be induced by glucose deficiency. Zhu et al. found that *HOXC-AS3* was involved in the deacetylation modification of histone H3 in a SIRT6-dependent manner. *HOXC-AS3* promoted the dissociation of SIRT6 from the promoters of key genes involved in energy metabolism by binding to SIRT6, thereby enhancing PDK4, LDHA, and PFK1 expression. In vitro and in vivo models constructed by the authors revealed that *HOXC-AS3* triggered a cellular metabolic paradigm shift resulting in enhanced proliferation, metastasis, and various other vital activities of tumor cells. This suggests that energy metabolic reprogramming affects various aspects of tumors [57].

The most critical regulator of glycolytic flow is the activity of PFK-1, which is influenced by various allosteric effectors. Intracellular allosteric regulator fructose-2,6-bisphosphate (F-2,6-BP) is a potent activator of PFK-1. F-2,6-BP increases the affinity of PFK-1 for F6P and allows glycolytic flux to be synthesized into F1,6BP [58]. The intracellular steady-state concentration of F-2,6-BP is controlled by the homodimeric bifunctional enzyme PFK-2/FBPase (PFKFB)family [59]. Research has shown that *circ_0102273* could upregulate PFKFB3 expression by sponging *miR-1236-3p*, which, in turn, promoted breast cancer proliferation, metastasis, and glycolysis [60]. Wang et al. found that PFKFB3 was a direct target of *tRiMetF31*, and knockdown of *tRiMetF31* restored PFKFB3-driven angiogenesis, with elevated PFKFB3 significantly correlating with metastasis [61].

PK catalyzes the final reaction in glycolysis, transferring high-energy phosphate from phosphoenolpyruvate (PEP) to ADP to produce ATP and pyruvate [62]. Mammals have four types of pyruvate kinase isoenzymes: the L and R isoforms expressed in the liver and red blood cells; the M1 subtype found in tissues requiring rapid energy production; and the M2 isoform, a splicing variant of M1 expressed during embryonic development [63]. Pyruvate kinase isoenzyme M2 (PKM2) is notably expressed in proliferative cells and tumor cells [64]. *CircKIF4A*, primarily enriched in the cytoplasm, could bind to *miR-335*, while ALDOA and OCT4 were downstream targets of *miR-335*. ALDOA and OCT4 were metabolism-related proteins that regulate glycolytic proteins like HK2 and PKM2 [65–67]. Elevated *miR-335* expression led to reduced ALDOA/HK2 and OCT4/PKM2 protein levels. Consequently, *circKIF4A* regulated glucose metabolism through the *miR-335*-ALDOA/OCT4-HK2/PKM2 axis [68]. Zheng et al. found that HIF-1α anti-sense lncRNA, *HIFAL*, was essential for maintaining and enhancing HIF-1α-mediated transactivation and glycolysis. Mechanistically, *HIFAL* recruit prolyl hydroxylase 3 (PHD3) to PKM2, inducing its prolyl hydroxylation and facilitating the entry of the PKM2/PHD3 complex into the nucleus by binding with heterogeneous nuclear ribonucleoprotein F (hnRNPF). This enhances HIF-1α transactivation and increases glucose uptake and lactate production in breast cancer cells [69].

In addition to the three enzymes mentioned above, phosphoglycerate kinase 1 (PGK1) plays a vital role in glycolysis as an essential enzyme for substrate-level phosphorylation. PGK1 catalyzes the transfer of phosphate groups on mixed anhydrides from carboxyl to ADP for producing 3-phosphoglycerate (3-PG) and ATP. Simultaneously, PGK1 promotes glucose uptake and lactate production in cancer cells. *MiR-16–1-3p* acts as an upstream regulator of PGK1, inhibiting PGK1 expression by targeting *PGK1* 3’-UTR. *MiR-16–1-3p* can inhibit tumor glycolysis by suppressing glucose uptake and lactate production, thereby decreasing ECAR and increasing cellular oxygen consumption rate (OCR) [70].

#### Lactate or acetyl-CoA

Whether pyruvate, a product of glycolysis, is reduced to lactate catalyzed by lactate dehydrogenase (LDHA) in the cytoplasm or enters the mitochondria to generate acetyl CoA catalyzed by the pyruvate dehydrogenase (PDH) complex, affects the direction of glycolysis. LDHA and PDH are positioned at the intersection of glycolysis and the citric acid cycle, serving as crucial enzymes bridging the oxygen-independent and dependent pathways.

LDHA catalyzes the conversion of pyruvate to lactic acid. As with the Warburg effect, most tumor cells exhibit heightened glycolysis and increased lactic acid production. Excessive lactate leads to extracellular acidosis, promoting invasion, angiogenesis, metastasis, and impacting the immune response, which tends to be associated with a poor prognosis [71]. Chen et al. found that in the nucleus, the *LncRNA DIO3OS* interacted with polypyrimidine tract binding protein 1 (PTBP1), a splicing repressor. *DIO3OS* and PTBP1 bind to and protect the 3'UTR of *LDHA* mRNA from splicing-induced deficiency which maintains its integrity and stability. This ultimately activates glycolytic metabolism in drug-resistant breast cancer cells, conferring a growth advantage to these cells [72]. Xing et al. demonstrated that *circPDCD11* could regulate LDHA levels by sponging *miR-432-5p*. Overexpression of *circPDCD11* plays an important role in promoting glucose uptake, lactic acid production, and ECAR. Clinical studies have also shown that high expression of *circPDCD11* is associated with a poor prognosis [73]. Similarly, Zan et al. found that *Circ-CSNK1G1* was overexpressed in triple-negative breast cancer, and its target *miR-28-5p* can inhibit breast tumor growth and metastasis by suppressing LDHA-induced glycolytic energy metabolism [74].

The PDH complex serves as the rate-limiting enzyme that links glycolysis and the TCA cycle. Pyruvate dehydrogenase protein X (PDHX) is a structural component of the PDH complex and is essential for its activity. Loss of PDH activity or function signifies the cellular transition to the glycolytic state, where pyruvate is converted to lactate in cancer cells, inducing aerobic glycolysis. Thus, PDHX (and PDH complexes in general) effectively exert tumor-suppressive effects by maintaining normal metabolic homeostasis [75]. Eastlack et al. found that *miR-27b* inhibited the expression of the PDH complex by targeting the 3'UTR of *PDHX*, resulting in specific metabolic dysregulation. Overexpression of *miR-27b* inhibits PDH function, leading to the accumulation of large amounts of pyruvate upstream, which is then directed into lactate production to sustain glycolytic flux or be used for cellular biosynthetic purposes. Ultimately, the metabolic consequences of this dysregulated interaction may contribute to poor prognosis in breast cancer patients [76].

#### Glycogen synthesis and catabolism

Glycogen is a glucose polymer and serves as the primary storage form of glucose. Glycogen synthesis and breakdown are primarily managed by glycogen synthase (GS) and glycogen phosphorylase (GP). GS enzymes extend the glycogen branch through the formation of α-1,4 glycosidic bonds, while GP enzymes break them down to produce glucose-1-phosphate (G1P). G1P can be further converted to G6P catalysed by phosphate glucose metastases (PGM), for various metabolic pathways. In addition, the dephosphorylation of G6P to free glucose requires glucose-6-phosphatase(G6P) [77, 78].

PGM5 is a member of the PGM superfamily and catalyzes the bidirectional interconversion of G1P and G6P. In a study by Ran et al., a comparison of breast cancer tissues with paraneoplastic tissues revealed that PGM5 levels are downregulated in breast cancer tissues. Cytological experiments demonstrated that overexpression of PGM5 inhibited proliferation, migration and epithelial-mesenchymal transition (EMT) of breast cancer cells. Subsequent database screening identified *miR-1224-3p* as an inhibitor of *PGM5* expression, directly targeting its 3' UTR. This, in turn, promoted the production of lactate, ATP, and G6P in breast cancer cells. The *miR-1224-3p/PGM5* axis was found to regulate breast cancer cell proliferation and migration through aerobic glycolysis [79].

Glucose-6-phosphatase catalytic (G6PC) subunit is one of the three genes that encode the catalytic subunit of glucose-6-phosphatase in humans. It serves as a key enzyme in glucose homeostasis, playing roles in gluconeogenesis and glycogenolysis. In their study on identifying differentially expressed tRFs in triple-negative breast cancer (TNBC) tissues, Zhu et al. discovered that the expression of *tRF*^*Lys−CTT−010*^ was significantly increased in tumor tissues and promoted the proliferation and migration of TNBC cells. To investigate the molecular mechanism, the authors conducted a KEGG analysis of differentially expressed genes associated with *tRF*^*Lys−CTT−010*^ and found multiple genes related to the amylose sucrose metabolic pathway. Furthermore, G6PC was identified as a potential target of *tRF*^*Lys−CTT−010*^. Overexpressing *tRF*^*Lys−CTT−010*^ significantly reduced the amount of glycogen in cells, and this effect was counteracted by G6PC knockdown. Therefore, *tRF*^*Lys−CTT−010*^ upregulates lactate and downregulates glycogen, reprogramming cancer glucose metabolism through G6PC, thereby affecting tumor progression [80].

**Table 1:** Non-coding RNAs regulating glucose metabolism in breast cancer

Pathway	Target enzyme	ncRNAs	Function	Refs.
Glucose transport	GLUT	*miR-122*	Downregulate	[81]
		*miR-186-3p*	Downregulate	[51]
		*LINC00346*	Upregulate	[48]
		*TSPEAR-AS2*	Upregulate	[44]
		*circ_0001955*	Upregulate	[45]
		*circ_0072995*	Upregulate	[46]
		*circTFF1*	Upregulate	[47]
Glycolysis	HK	*miR-155*	Upregulate	[43]
		*circDENND4C*	Upregulate	[82]
		*circYY1*	Upregulate	[83]
		*circ_0008039*	Upregulate	[84]
		*circWHSC1*	Upregulate	[85]
		*circ_0069094*	Upregulate	[86]
		*circ_0000442*	Downregulate	[87]
		*circ_0001982*	Upregulate	[88]
		*circRNF20*	Upregulate	[89]
	PFKFB	*circ_0102273*	Upregulate	[60]
		*tRiMetF31*	Downregulate	[61]
		*BCAR4*	Upregulate	[90]
		*circCARM1*	Upregulate	[91]
	PFK1	*HOXC-AS3*	Upregulate	[57]
		*SNHG5*	Upregulate	[55]
	PGK1	*miR-16-1-3p*	Downregulate	[70]
	PKM2	*HIFAL*	Upregulate	[69]
		*FGD5-AS1*	Upregulate	[92]
		*circKIF4A*	Upregulate	[68]
Lactate or acetyl-CoA	LDHA	*DIO3OS*	Upregulate	[72]
		*miR-34a*	Downregulate	[93]
		*circPDCD11*	Upregulate	[73]
		*circKLHL24*	Downregulate	[94]
		*circCSNK1G1*	Upregulate	[74]
		*circABCB10*	Upregulate	[95]
	PDHX	*miR-27b*	Downregulate	[76]
Glycogen synthesis and catabolism	PGM5	*miR-1224-3p*	Downregulate	[79]
	G6PC	*tRF*^*Lys−CTT−010*^	Upregulate	[80]

## Non-coding RNA and breast cancer lipid metabolism

**Fig. 3:**
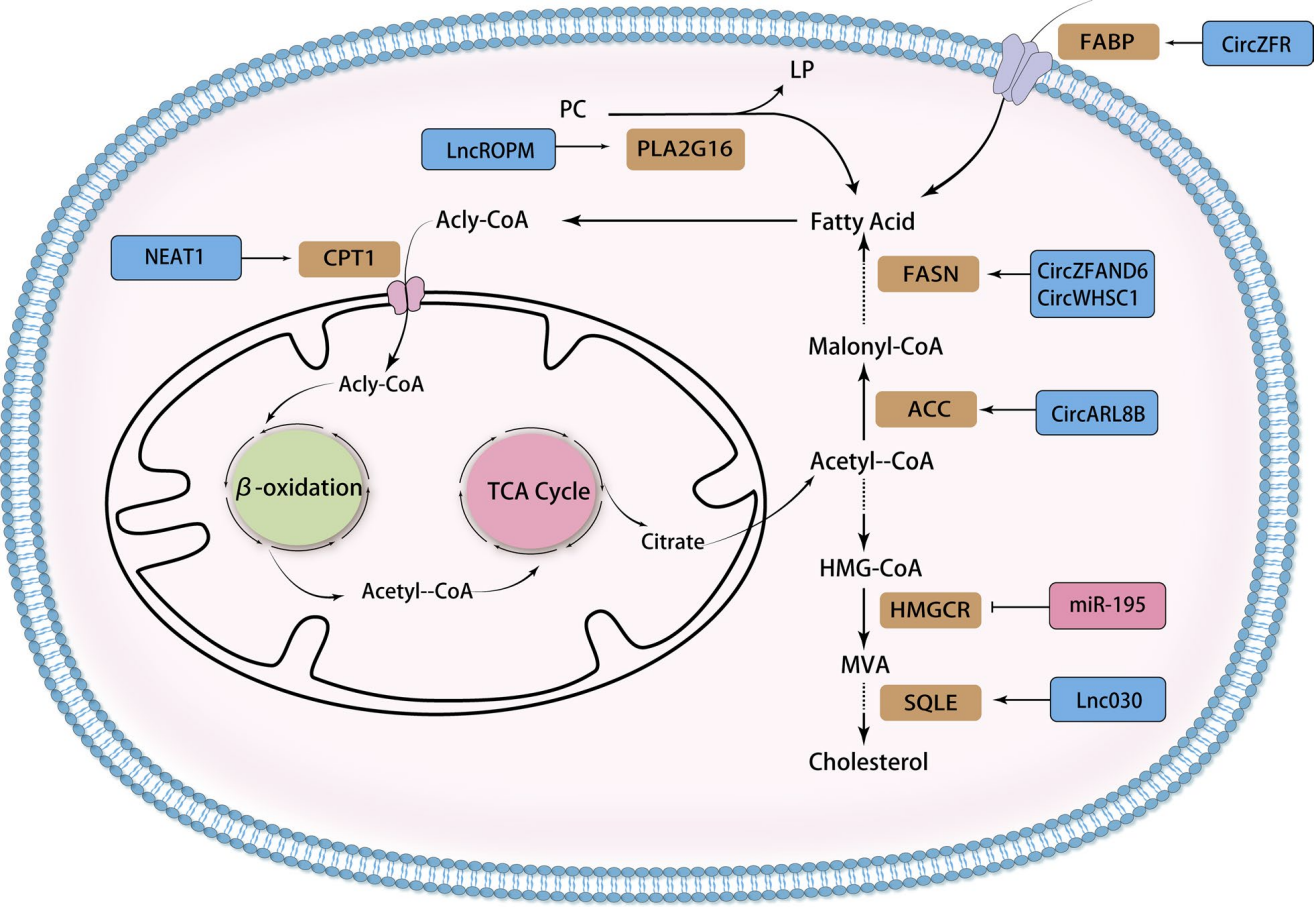
Steps in ncRNA-regulated reprogramming of fatty acid synthesis, β-oxidation, cholesterol synthesis and phospholipid metabolism in breast cancer. Blue boxes and arrows represent positive regulations, while red boxes and lines with blunt ends represent negative regulations. Dashed lines indicate omitted steps

Tumor cells rely on a continuous supply of fatty acids for their growth and proliferation. Most normal cells, in other words cells that do not undergo lipid synthesis, take up lipids from the extracellular environment mainly through exogenous fatty acids circulating in the plasma. In contrast, lipogenic normal cells, mainly hepatocytes, tend to activate the de novo fatty acid biosynthetic pathway under energetically adequate conditions. This pathway converts glucose-derived acetyl coenzyme into lipids that can be stored in adipose tissue. Notably, most cancer cells exhibit an 'adipogenic phenotype', which means that the de novo fatty acid biosynthetic pathway is enhanced, independent of extracellular lipid levels. Non-coding RNAs influence biological processes related to fatty acid transport processes, fatty acid synthesis and catabolism, and cholesterol synthesis by regulating the expression of a range of lipid metabolism-related enzymes (Fig. 3; Table 2).

### Fatty acid transport

Fatty acid binding proteins (FABPs) facilitate the transport of fatty acids to different organelles and regulate their metabolism, as well as other physiological activities. Among these, FABP7, known as brain-type FABP, is considered an important molecule for cell proliferation in healthy and diseased organisms. Elevated levels of FABP7 have been reported to correlate with poor prognosis in various types of tumors [96]. Tian et al. uncovered that *circ_ZFR* acted as a tumor promoter in breast cancer. When exploring the mechanism of *circ_ZFR* action, they found that *circ_ZFR* promoted breast cancer cell progression by regulating the *miR-223-3p*/FABP7 axis. Specifically, *circ_ZFR* could promote BC cell proliferation, migration, invasion and EMT while suppress apoptosis by serving as a sponge for *miR-223-3p* and regulating FABP7 [97].

### Fatty acid synthesis and degradation

Fatty acid synthesis is orchestrated by several enzymes constituting the fatty acid synthase complex. Among these, acetyl CoA carboxylase (ACC) is a pivotal enzyme in fatty acid synthesis, catalyzing the initial step. ACC converts acetyl CoA into malonic acid monoacyl CoA, providing the essential raw material for fatty acid synthesis. In breast cancer cells, it was found that *circARL8B* influenced the expression levels of ACC1, FASN, and FABP5. Silencing *circARL8B* led to reduced cellular levels of phospholipids, triglycerides, and various lipid-metabolizing enzymes. The mechanism of action of circARL8B revealed that its silencing decelerated BC cell growth, metastasis, and fatty acid metabolism by regulating the *miR-653-5p*/HMGA2 axis [98].

Fatty acid synthase (FASN) is a key enzyme in the endogenous lipogenic pathway, responsible for catalyzing the synthesis of long-chain saturated fatty acids from acetyl coenzyme A and malonyl coenzyme A, primarily utilizing NADPH as a reducing agent [99]. FASN activation is an early and nearly universal hallmark of most human cancers or their precursor lesions, and increases in a stage-dependent manner, correlating with worsening patient survival. The relationship between FASN status and prognosis strongly suggests that FASN-catalyzed endogenous adipogenesis provides a growth and survival advantage to cancer cells [100]. *CircZFAND6* acted as a ceRNA by sponging *miR-647*, resulting in increased FASN expression that promoted breast cancer proliferation and metastasis [101]. Similarly, *circWHSC1* bound to and inhibited the expression of *miR-195-5p* via sponge action, with FASN being a downstream target of *miR-195-5p*. Thus, the *CircWHSC1 /miR-195-5p*/FASN axis was present in breast cancer cells to influence breast cancer progression. FASN enhanced proliferation and metastasis of breast cancer cells by attenuating the activation of AMPK/mTOR pathway, thereby impacting the sensitivity of chemotherapy or radiotherapy for breast cancer [85].

When oxygen supply is sufficient, fatty acids undergo four stages of fatty acid activation, transfer to mitochondria, β-oxidation to generate acetyl CoA, and subsequent entry into the tricarboxylic acid cycle, leading to the release of a substantial amount of ATP. Fatty acid oxidation (FAO) is a major pathway that regulates fatty acid degradation and promotes ATP and NADPH production. FAO-mediated mitochondrial bioenergetics is thought to play a key role in cell proliferation, cancer stemness and chemoresistance [102]. The carnitine shuttle system is the rate-limiting step in FAO. An essential component of this system is carnitine palmitoyltransferase 1 (CPT-1), situated on the outer mitochondrial membrane. CPT-1 facilitates the formation of the acylcarnitine complex, which is then able to cross the inner mitochondrial membrane via other members of the shuttle system and eventually undergo β-oxidation [103]. CPT1A, a subtype of the CPT1 gene family, is expressed in all tissue types except skeletal muscle cells and brown adipocytes. Xiong et al. found that *miR-107* negatively regulated breast cancer cell progression by targeting genes related to tumor development, including CPT1A. Notably, the regulation of CPT1A by *miR-107* can also be mediated through LncRNA nuclear paraspeckle assembly transcript 1 (NEAT1). *LncRNA NEAT1* negatively regulated *miR-107,* promoting CPT1A expression and facilitating β-oxidation, ultimately contributing to tumor progression [104].

### Cholesterol synthesis

Cholesterol is an essential component of cell membranes and a precursor to steroid hormones, bile acids, vitamin D and oxysterols, key molecules for cell growth and function [105]. Cholesterol synthesis relies on acetyl-CoA as its fundamental building block. Acetyl-CoA is produced through the breakdown of glucose, amino acids, and fatty acids in the mitochondria. However, it cannot directly traverse the inner mitochondrial membrane. To become accessible, it must first combine with oxaloacetic acid in the mitochondria to form citric acid. This citric acid then passes into the cytoplasm through a carrier in the inner mitochondrial membrane, where it is cleaved into acetyl-CoA–a key ingredient in cholesterol synthesis. Upregulation of cholesterol synthesis plays a supportive role in tumor growth, metastasis, stemness, and treatment resistance. This phenomenon extends to breast cancer, where clinical evidence indicates that cholesterol and its metabolites promote tumor progression [106]. Huang et al. identify differentially expressed tRF in normal and breast cancer cell lines. Among these, *tDR-4733* was found to target genes involved in lipid metabolism, encompassing steroid metabolism, lipid biosynthesis, bile acid metabolism, and bile acid biosynthesis processes. Since cholesterol is an indispensable precursor of steroid hormones, it's plausible that *tDR-4733* may facilitate breast cancer development by disrupting cholesterol metabolism [107].

Cholesterol synthesis is a multifaceted process divided into three primary stages. In the first stage, three molecules of acetyl-CoA are transformed into HMG-CoA, catalyzed by sulfurylase and 3-hydroxy-3-methylglutaryl CoA (HMG-CoA) synthase. In the second stage, HMG-CoA is converted into mevalonate (MVA) by HMG-CoA reductase (HMGCR). The third stage involves a series of phosphorylation, decarboxylation, dehydroxylation, and condensation reactions that ultimately lead to the formation of squalene, facilitated by endoplasmic reticulum cyclase and oxygenase. This squalene is then transformed into cholesterol through a multistep process involving redox reactions.

HMGCR is a key enzyme in the synthesis of cholesterol, catalyzing the conversion of HMG-CoA into MVA. Singh et al. found that overexpression of *miR-195* downregulated the expression of HMGCR, ACACA, and FASN. Consequently, this decrease resulted in lower cholesterol and triglyceride levels. The findings illustrate that *miR-195* targets genes associated with adipogenesis and cholesterol synthesis, thereby mitigating the epithelial-mesenchymal transition in breast cancer cells., opening up a new target for breast cancer treatment [108].

Squalene epoxidase (SQLE) catalyzes the first oxygenation step in the conversion of squalene to 2,3(S)-monoxysqualene (MOS) in phase III of cholesterol synthesis [109]. SQLE plays an oncogenic role in a variety of tumors, including breast cancer. High expression of SQLE can be observed in a variety of cancers and is associated with tumor metastasis and patient prognosis [110, 111]. In a study conducted by Qin et al., it was revealed that *Lnc030* promoted cholesterol synthesis by modulating the stability of *SQLE* mRNA through its interaction with poly(rC)-binding protein 2 (PCBP2). This interaction created an axis involving *Lnc030*, SQLE, and cholesterol, which in turn triggered the activation of the PI3K/Akt signaling pathway. This pathway was associated with maintaining the stemness of breast cancer stem cells (BCSCs) and promoted breast tumorigenesis and growth [112].

### Phospholipid metabolism

Phosphatidylcholine is the predominant phospholipid found in eukaryotic cell membranes, and it plays a crucial role in cell proliferation, differentiation, and the maintenance of normal cell cycle regulation. Various phospholipases are involved in the degradation of glycerophospholipids within organisms, acting on different ester bonds of these phospholipid molecules to facilitate their degradation. Among these enzymes, PLA2G16 is responsible for the efficient release of free fatty acids (FFAs) and lysophospholipids (LP) from phosphatidylcholine (PC) [113]. The authors found that *LncROPM* could enhance the stability of *PLA2G16* by binding to its 3'-UTR region. Two metabolic substrates of PLA2G16, phosphatidylcholine (PC) and glycerophosphoglycerol(PG), were significantly elevated in *lncROPM* down-regulated BCSC. Elevated PLA2G16 significantly promoted phospholipid metabolism and free fatty acid production, especially arachidonic acid [114]. Arachidonic acid activation, in turn, triggered the PI3K/AKT signaling pathway, and this activation was closely related to the maintenance of stemness in BCSCs. The activated PI3K signaling had regulatory control over arachidonic acid metabolism. This suggests the presence of a positive feedback loop between the PI3K signaling pathway and arachidonic acid, further promoting the malignant behavior of certain cancer cells [115].

**Table 2:** Non-coding RNAs regulating lipid metabolism in breast cancer

Pathway	Target enzyme	ncRNAs	Function	Refs.
Fatty acid transport	FABP	*Circ_ZFR*	Upregulate	[97]
Fatty acid synthesis and degradation	ACC	*circARL8B*	Upregulate	[98]
	FASN	*circZFAND6*	Upregulate	[101]
		*circWHSC1*	Upregulate	[85]
	CPT-1	*NEAT1*	Upregulate	[116]
Cholesterol synthesis	HMGCR	*miR-195*	Downregulate	[108]
	SQLE	*Lnc030*	Upregulate	[112]
Phospholipid metabolism	PLA2G16	*LncROPM*	Upregulate	[115]

## Non-coding RNA and breast cancer amino acid metabolism

**Fig. 4:**
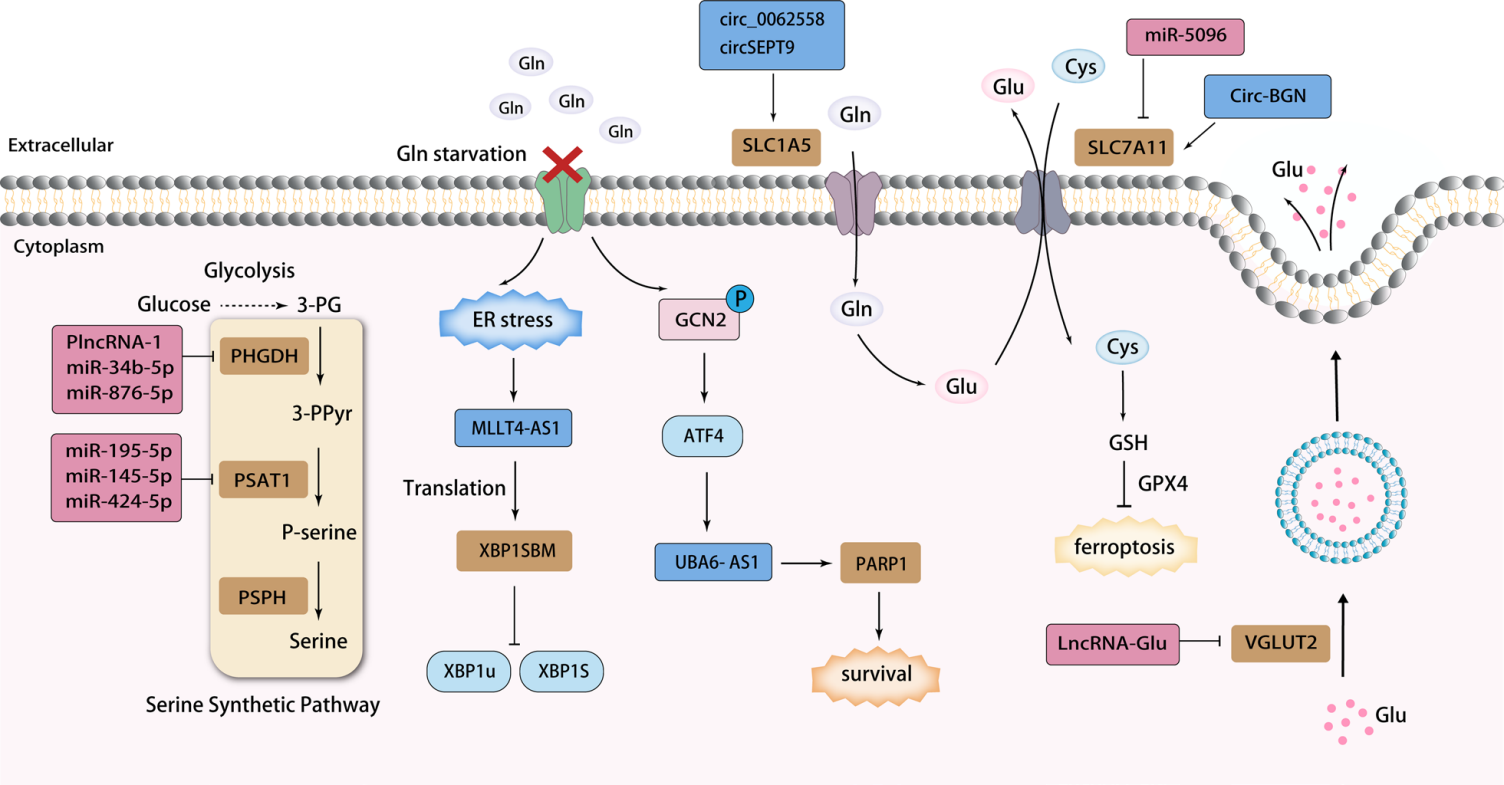
Steps in ncRNA-regulated reprogramming of amino acid metabolism. Blue boxes and arrows represent positive regulations, while red boxes and lines with blunt ends represent negative regulations

Amino acids are critical components in the rapid growth of cancer cells, serving as substrates for protein synthesis. Amino acids can be categorized into two groups based on whether the body can synthesize them: essential amino acids and non-essential amino acids. Tumor cells often lose the expression of enzymes involved in the synthesis of non-essential amino acids through direct mutation or silencing, thus affecting amino acid utilization [117]. Non-coding RNAs are also involved in this process of regulating amino acid metabolism (Fig. 4; Table 3).

### Amino acid transport

Maintaining high levels of glutamine in the blood provides a ready source of carbon and nitrogen to support biosynthesis, energetics and cellular homeostasis, which cancer cells may exploit to fuel tumor growth. Glutamine enters cells via transporters, such as solute carrier family 1 neutral amino acid transporter member 5 (SLC1A5). Glutamine-derived glutamate can also be exchanged for extracellular cystine via the SLC7A11 transporter protein, which exchanges intracellular glutamate for extracellular cystine [9]. Yuan et al. found that *circ_0062558* could function as a tumor promoter in TNBC through inhibition of *miR-876-3p*. Knockdown of *circ_0062558* resulted in reduced relative glutamine depletion, relative α-ketoglutarate production and relative glutamate production in BC cells. SLC1A5 was bioinformatically predicted to be a putative target of *miR-876-3p*, while *miR-876-3p* enrichment was verified to significantly inhibit SLC1A5 protein expression. Thus. *circ_0062558* could enhance glutamine transport through the *miR-876-3p*/SLC1A5 axis to support the energy requirements for breast cancer development [118]. Likewise, Wang et al. have demonstrated that *circSEPT9* regulated breast cancer progression through the *miR-149-5p*/SLC1A5 pathway, and the downregulation of SLC1A5 impeded glutamine uptake, cell proliferation and induced apoptosis in BC cells [119].

Cystine/glutamate anti-transporter SLC7A11 promotes tumor growth not only through the import of cystine for glutathione biosynthesis and antioxidant defence, but also by suppressing ferroptosis. Ferroptosis is an iron-dependent form of cell death characterized by significant iron accumulation and lipid peroxidation [120]. Cells have evolved a variety of mechanisms to defend against these toxic lipid peroxides, with glutathione peroxidase 4 (GPX4) playing a prominent role. Members of the GPX family inhibit iron death by using reduced glutathione (GSH) as a cofactor to detoxify lipid peroxides to lipidsol. Cysteine is the rate-limiting precursor for glutathione synthesis, and intracellular cysteine is mainly supplied by SLC7A11-mediated cystine uptake [121]. This is the mechanism by which SLC7A11 inhibits the onset of ferroptosis. In Yadav's study, SLC7A11 overexpression was significantly associated with poor survival in breast cancer patients. The authors identified and validated that *miR-5096* could target the 3'UTR of *SLC7A11* to downregulate its expression and induce cell death. Importantly, the study found that blockade of cysteine transport by inhibition of SLC7A11 was not the only mechanism observed for *miR-5096*-mediated cell death, but was also associated with ferroptosis. SLC7A11 played a key role in *miR-5096*-mediated ferroptosis, which induces changes in ROS, hydroxyl radicals, and lipid peroxides, which are all are known drivers of ferroptosis [122]. Wang et al. Discovered that *Circ-BGN* can directly bind to SLC7A11 and OTUB1, a deubiquitinating enzyme that increases SLC7A11 stability. Therefore, *Circ-BGN* enhances OTUB1-mediated deubiquitination of SLC7A11 and upregulates SLC7A11 protein expression to inhibit ferroptosis [123].

In addition to the transporter approach, glutamate can also be transported through vesicular structures. Vesicular glutamate transporter 2 (VGLUT2) is a key vesicular protein involved in the transport of glutamate into synaptic vesicles for cytosolic release [124, 125]. Yin et al. found that *lncRNA-Glu* overexpression reduced glutamate uptake at the vesicular membrane by glutamate uptake assays, demonstrating that binding of *lncRNA-Glu* to VGLUT2 reduced the glutamate transporter activity of VGLUT2 [126]. It is well known that glutamate is a neurotransmitter, and its translocation to the extracellular compartment can lead to a series of biological effects, ultimately contributing to the invasive behavior of tumor cells.

### Glutamine addiction

As an essential metabolic resource, glutamine is involved in a variety of energy-forming and metabolic pathways, including the replenishment of the tricarboxylic acid cycle and the biosynthesis of nucleotides, GSH and other non-essential amino acids. Therefore, glutamine deprivation inhibits cancer growth and even induction of cell death [127]. This metabolic dependence of transformed cells on glutamine constitutes what has recently been defined as glutamine addiction [128]. Wu et al. observe that *LncRNA UBA6-AS1* expression is upregulated in various breast cancer cell lines deprived of glutamine or arginine, two key amino acids required for cancer cell growth. To explore the role of *UBA6-AS1* in metabolic stress, it was found that inhibition of *UBA6-AS1* reduced cell viability under amino acid deprivation treatment, suggesting that *UBA6-AS1* was protecting cells from metabolic stress-induced cell death. In examining the upstream and downstream regulatory mechanisms involved in *UBA6-AS1*, it is found that *UBA6-AS1* is differentially regulated by the GCN2/ATF4 signaling pathway when Gln or Arg is deprived. PARP1 is found to be a downstream target of *UBA6- AS1* and is regulated by the GCN2/*UBA6-AS1* axis, suggesting that PARP1 is also involved in metabolic stress. In addition, PARP1 has been shown to promote migration or invasion by regulating promoter activity or the expression of invasiveness-related genes [129].

A study by Wu et al. found that TNBC were more sensitive to Gln deficiency compared to non-TNBC. The researchers identified a differentially expressed LncRNA named *MLLT4-AS1* when specifically looking at lncRNAs associated with Gln starvation in TNBC. While lncRNAs were traditionally thought to lack protein-coding capacity, recent studies have shown that some lncRNAs can encode functional micropeptides [130]. In this study, the authors discovered that *MLLT4-AS1* encoded a 21 amino acid micropeptide named “XBP1s binding micropeptide” (XBP1SBM). XBP1SBM was significantly upregulated in TNBC cell lines under Gln starvation but not in non-TNBC cell lines. Gln starvation induced an endoplasmic reticulum stress response in TNBC, which in turn activates the IRE1α-XBP1 pathway. This pathway promoted the transcription of *MLLT4-AS1* and increased the translation of XBP1SBM. XBP1SBM was found to disrupt the interaction between XBP1u and XBP1s, leading to a significant change in the subcellular localization of XBP1 and an increase in nuclear XBP1. Consequently, this led to enhanced VEGF transcription. Ultimately, in TNBC cells, Gln starvation-induced XBP1SBM resulted in a significant increase in intracellular and secreted VEGF, promoting angiogenesis and metastasis [131].

### Serine biosynthesis

Serine synthetic pathway (SSP), as an important branch of the glycolytic pathway, controls the flux of glycolytic intermediates into serine and its downstream substances. SSP converts 3-phosphoglyceric acid generated by glycolysis into serine through the action of phosphoglycerate dehydrogenase(PHGDH), phosphoserine aminotransferase(PSAT) and phospho serine phosphatase (PSPH). PHGDH, the enzyme that catalyzes the first step of the serine biosynthesis pathway, is elevated in 70% of estrogen receptor (ER)-negative breast cancers [132]. Li et al. found that LncRNA PlncRNA-1 overexpression inhibited the growth of breast cancer by upregulating TGF-β1 and downregulating PHGDH [133].

PSAT is another key enzyme in the serine synthesis pathway, which catalyzes the conversion of 3-phosphohydroxypyruvate(3-PPyr) to phosphoserine(p-serine). P-serine can further generate serine and glycine, which are involved in downstream one-carbon metabolism and nucleic acid metabolism [134]. PSAT1 also catalyzes the generation of α-ketoglutarate (α-KG) from glutamate, which enters the tricarboxylic acid cycle. This participation in complex metabolic networks provides tumor cells with the necessary material and energy for proliferation while maintaining intracellular redox balance. Wang et al. found that *miR-195-5p* exerts anti-tumor effects in TNBC cells by targeting *PSAT1* [135]. Additionally, Petri identified four miRNAs in breast cancer cells that directly target the *PSAT1* 3'UTR (*miR-145-5p* and *miR-424-5p*) and the *PHGDH* 3'UTR (*miR-34b-5p* and *miR-876-5p*). Increased expression of these miRNAs restored the sensitivity of endocrine therapy for breast cancer [136]. It's evident that non-coding RNAs can act as tumor suppressors by targeting enzymes that are crucial in serine biosynthesis.

As discussed above, it is evident that the metabolism of glucose, lipids, and proteins in the body is interconnected rather than isolated. These metabolic processes are linked and transformed through common intermediate metabolites, such as the tricarboxylic acid cycle and biological oxidation. Glucose undergoes catabolism via glycolysis, leading to the production of pyruvate. Subsequently, pyruvate is converted into acetyl CoA, which can enter the tricarboxylic acid cycle to generate energy. Acetyl CoA can also be carboxylated to form malonyl CoA, a precursor for synthesizing fatty acids and fats. Furthermore, intermediate products resulting from glucose metabolism, such as pyruvate and α-ketoglutarate, can be aminated to produce specific non-essential amino acids. Moreover, acetyl CoA, generated through the catabolism of amino acids, can be utilized for the synthesis of fatty acids and cholesterol. As a result, the body's metabolism of various nutrients remains in a dynamic balance. Non-coding RNAs play a crucial regulatory role in these intricate metabolic networks. Targeting non-coding RNAs to modulate metabolism can have a profound effect on the metabolic state of tumors, ultimately influencing tumor growth and suppression.

**Table 3:** Non-coding RNAs regulating amino acid metabolism in breast cancer

Pathway	Target enzyme	ncRNAs	Function	Refs.
Amino acid transport	SLC1A5	*Circ_0068255*	Upregulate	[118]
		*CircSEPT9*	Upregulate	[119]
	SLC7A11	*miR-5096*	Downregulate	[122]
		*Circ-BGN*	Upregulate	[123]
	VGLUT2	*LncRNA-Glu*	Downregulate	[126]
Serine biosynthesis	PHGDH	*PlncRNA-1*	Downregulate	[133]
		*miR-34b-5p*	Downregulate	[136]
		*miR-876-5p*	Downregulate	[136]
	PSAT	*miR-195-5p*	Downregulate	[135]
		*miR-145-5p*	Downregulate	[136]
		*miR-424-5p*	Downregulate	[136]

## Clinical implications of metabolism-regulating ncRNAs in breast cancer

In the current management of breast cancer, there are several tasks and challenges. These include the identification of molecular markers with optimal specificity and sensitivity for use in breast cancer screening, diagnosis, and prognosis. Additionally, the search for new therapeutic targets with clinical applicability is an important task. Enhancing the sensitivity of existing therapies is another priority, as well as finding ideal sensitizers or alternative treatments that do not entail long-term toxicity. In the following, we will describe the application of metabolism-regulating ncRNAs to influence existing therapeutic sensitivities and as new therapeutic targets and biomarkers.

### Modulation of resistance to existing therapeutic approaches

At present, breast cancer is managed through a combination of endocrine therapy, targeted therapy, chemotherapy, along with radiotherapy and immunotherapy, all tailored to the molecular characteristics of the patient's tumor and its treatment sensitivity. The occurrence of metabolic reprogramming can cause drug resistance or therapeutic resistance in breast cancer, which ultimately leads to poor prognosis. Non-coding RNAs can regulate this process by targeting metabolism-related genes to restore metabolic homeostasis, thereby increasing tumor sensitivity to drugs and other therapeutic approaches.

#### Endocrine therapy

Patients with invasive breast cancer positive for hormone receptors, such as ER and/or PR, typically require adjuvant post-operative endocrine therapy [44]. In premenopausal breast cancer patients, tamoxifen is the preferred choice for endocrine therapy. Nevertheless, the reprogramming of breast cancer cell metabolism has been associated with the development of tamoxifen resistance [137]. Tamoxifen-resistant breast cancer cells exhibit hyperactivation of glucose metabolism, including increased glucose transport and overactive glycolysis. Knockdown of glucose transport proteins or inhibition of glycolytic enzyme activity in resistant breast cancer cells can sensitize these cells to tamoxifen once again [138–140]. In the context of tamoxifen resistance, a study by He et al. revealed that *miR-186-3p* level was significantly reduced in tamoxifen-treated breast cancer cells. As mentioned above, *miR-186-3p* inhibited aerobic glycolysis by targeting EREG and acted as an important regulator of glycolysis and tamoxifen resistance in ER-positive breast cancer cells and tumors. Animal experiments demonstrated the therapeutic effect of 2′-*O*-methyl-modified *miR-186-3p* (agomiR-186-3p) on tamoxifen-resistant breast tumors, supporting the miRNA could be a novel candidate target for therapeutic intervention in endocrine therapy-resistant breast tumors [51]. Not only does glucose metabolism come into play, but also phospholipid metabolism plays a role in endocrine therapy. *LncROPM* participated in the maintenance of stemness in breast cancer stem cells by modulating PLA2G16-mediated phospholipid metabolism. This ultimately conferred resistance of tumor stem cells to clinical therapeutic agents like tamoxifen [115]. In another study, Petri and colleagues discovered that increased expression of two key enzymes in the serine synthesis pathway, PSAT1 and PHGDH, was associated with poor prognosis in patients treated with tamoxifen. The authors identified four miRNAs that directly target the *PSAT1* 3'UTR (*miR-145-5p* and *miR-424-5p*) and the *PHGDH* 3'UTR (*miR-34b-5p* and *miR-876-5p*). Transient transfection of these miRNAs was shown to restore sensitivity to endocrine therapy in endocrine-resistant cells [136].

In postmenopausal patients, third-generation aromatase inhibitors (AIs) drugs have become the first-line adjuvant therapy for ER-positive breast cancer [2, 141, 142]. AIs inactivate the aromatase enzyme and quantitatively block the conversion of androgens to estrogens, thereby reducing estrogen levels and inhibiting the proliferation of ER-positive breast cancer cells [143]. Recent findings by Chen and colleagues highlight that AI-resistant breast cancer cells may rely more on aerobic glycolysis to drive tumor growth. They discovered that the *LncRNA DIO3OS* enhanced aerobic glycolysis by regulating the splicing switch, thereby conferring a growth advantage to AI-resistant cells. These results emphasize the critical role of *DIO3OS* in inducing AI resistance by activating an ER-independent proliferative pathway [72].

#### Targeted therapies

Targeted therapy for HER2-positive breast cancer patients is available with monoclonal antibodies that target this receptor, such as the monoclonal antibodies trastuzumab and patuximab. However, primary and acquired resistance occurring in HER2 + patients often leads to poor prognosis such as recurrence and metastasis [144]. Wang et al. identified *circ-BGN* as a key factor in trastuzumab resistance. *Circ-BGN* was significantly elevated in trastuzumab-resistant breast cancer cells and tissues and was associated with poorer overall survival. As previously described, *circ-BGN*'s mechanism of action involved enhancing OTUB1-mediated deubiquitination of SLC7A11, which inhibited ferroptosis. The ferroptosis inducer erastin was effective in restoring the antitumor effects of trastuzumab, which was more pronounced after co-knockdown of *circ-BGN* [123]. Trastuzumab-resistant cell manifestations also exhibit increased glycolysis and targeted therapy combined with glycolysis inhibitors may offer a promising anti-tumor strategy [145, 146]. Further exploration is needed to understand the role of non-coding RNAs in regulating this process.

#### Chemotherapeutic

In the treatment of TNBC, paclitaxel (PTX)-based regimens have proven to be a vital chemotherapeutic approach. However, the utility of PTX-based chemotherapy in TNBC is limited by PTX resistance. Huang et al. evaluated the role of *circWAC* in regulating glycolytic metabolism in BC cells. *CircWAC* interference inhibited glucose uptake, lactate production, and decreased the levels of GLUT1, LDHA, and HK2, thereby inhibiting glycolysis in BC cells [147]. Wang and his team demonstrated that downregulation of *circWAC* increased the sensitivity of TNBC cells to PTX in both cellular and animal modeling results [148]. Additionally, Park et al. found that deficiency of *LncRNA NEAT1* severely impairs breast cancer development, growth, and metastasis, specifically shutting down the penultimate step of glycolysis. *NEAT1*-regulated PGK1/PGAM1/ENO1 multiactivator complex, an unidentified "metabolic factor", functions in the second step of glycolysis to achieve efficient glycolysis through the substrate channel [149]. The exosomal *NEAT1* derived from BC cells induces resistance to paclitaxel in recipient cells. Inhibition of *NEAT1* expression with small hairpin RNA (shRNAs) improves paclitaxel response in BC patients [116]. These studies emphasize that non-coding RNAs can enhance chemosensitivity by modulating gluconeogenesis, whereas less research exists on the role of modulating lipid or amino acid metabolism.

#### Immunotherapy

Immunotherapy has emerged as an indispensable force in the treatment of tumors, harnessing the potential of the immune system for innovative treatment strategies. Immunotherapy encompasses a variety of approaches, including adoptive cell therapies, over-the-counter cellular therapies, vaccines, oncolytic viruses, and most notably, immune checkpoint blockade. FDA-approved immune checkpoint inhibitors like cytotoxic T-lymphocyte-associated antigen-4 (CTLA-4) inhibitors, programmed cell death receptor-1 (PD-1) inhibitors, and programmed cell death ligand-1 (PD-L1) inhibitors have proven effective in treating solid tumors [150]. Limiting metabolic competition in the tumor microenvironment (TME) may improve the effectiveness of immunotherapy. High glucose consumption by tumor cells often deprives T cells in the TME of vital nutrients. By diminishing the glucose competition from tumor cells, the therapeutic activity of CTLA-4 inhibitors can be enhanced. Hence, combining CTLA-4 inhibitors with glycolysis inhibitors may enhance their therapeutic potential [151]. For instance, in colorectal cancer, *CircQSOX1* was found to activate glycolysis in colorectal cancer cells, fostering immune evasion and ultimately impeding the response to anti-CTLA-4 therapy in colorectal cancer patients [152]. Additionally, silencing *microRNA-126* was found to significantly reduce CTLA-4 expression in breast cancer, ultimately leading to a reduction in the induction and suppression of Tregs [153].

We have known that high expression of PD-L1 in tumor cells contributes to tumor immune escape. It has been found that targeting aerobic glycolysis and amino acid metabolism in tumor cells can modulates PD-L1 expression [154]. For example, combination therapy with HK inhibitors and anti-PD-1 antibodies provides greater tumor suppression than each drug alone [155]. Huang et al. found that both *PDL1* and *LDHA* were target genes of *miR-34a* in TNBC. They observed a positive correlation between LDHA and PD-L1 expression. By competing for miR-34a, the *PDL1* 3’UTR and *LDHA* 3’UTR acted as ceRNAs to promote the expression and function of each other in TNBC. Consequently, a potential strategy for TNBC treatment could involve the concurrent targeting of PD-L1 and LDHA, combined with immunotherapy and metabolism-targeted therapy [156].

#### Radiotherapy

Radiation therapy is an integral component of comprehensive breast cancer treatment. It functions by either directly inducing DNA damage or indirectly triggering the production of reactive oxygen species (ROS) in cancer cells [157]. Radiotherapy resistance is a common hurdle in breast cancer treatment, often resulting in less favorable outcomes. Notably, resistance to radiotherapy has been linked to metabolic reprogramming, especially alterations in glucose metabolism [158]. Comparison of radiosensitized and radiation-resistant cancer cells revealed elevated rates of glycolysis and increased glucose uptake and lactate production in radioresistant cells [159].

It was observed that miR-34a plays a role in the response of breast cancer cells to DNA damage caused by low-energy X-rays. Notably, *miR-34a* expression displayed a negative correlation with radioresistance [160]. In a different context, *miR-34a* overexpression in hepatocellular carcinoma was shown to reduce glycolysis rates by inhibiting LDHA. This action led to resensitization of radio-resistant hepatocellular carcinoma cells to radiation therapy [159]. In the case of breast cancer, *miR-34a* was also found to suppress LDHA expression [93, 156], suggesting a potential mechanism for enhancing the sensitivity of breast cancer cells to radiation therapy by inhibiting glucose metabolism. Similarly, *miR-200c* is considered a radiosensitizer, with its expression showing a positive correlation with radiosensitivity [161]. In breast cancer, the upregulation of *miR-200c* was associated with the inhibition of lactate production and reduced HK2 protein levels [82]. Hence, non-coding RNAs can impact radiosensitization by modulating glycolysis rates. Furthermore, *circABCB10* was identified as a negative regulator of glycolysis in breast cancer through the *miR-223-3p*/PFN axis. Knockdown of *circABCB10* led to decreased protein levels of glycolysis-related factors, such as HIF1a, HK2, and LDHA, resulting in enhanced radiosensitivity of breast cancer cells [95]. Therefore, *circABCB10* inhibitors may serve as effective tools for targeting metabolic genes to act as radiosensitizers.

### Metabolism-regulating ncRNAs as therapeutic targets and biomarkers

#### Therapeutic targets

During tumorigenesis and progression, aberrant expression of ncRNAs is closely related to metabolic abnormalities. Therefore, targeting ncRNAs has emerged as a therapeutic tool to reshape the metabolic profile of tumor cells, resulting in therapeutic benefits. Therapies targeting tumor metabolism are an emerging therapeutic strategy to inhibit tumor growth and spread by interfering with the metabolic pathways of tumor cells [162]. By combining targeted ncRNA therapies with metabolic interventions, we can achieve more precise modulation of tumor cell metabolism and the functions of ncRNAs, thereby enhancing therapeutic outcomes.

Developing effective therapeutic strategies for manipulating ncRNAs targeting oncogenes or tumor suppressors is crucial to influence the activity of metabolism-related genes, thereby impacting tumor development. To enhance the in vivo stability and affinity of RNA drugs, chemical modifications are often applied. Common modifications include locked nucleic acid (LNA) and 2′-OH substitutions in place of ribose. The delivery of RNA drugs in vivo is primarily achieved using lipid-based nanocarriers, but recent advances have also introduced peptide and polymer delivery systems [163]. For miRNA targeting that requires overexpression, synthetic oligonucleotides consisting of miRNA duplexes (miRNA mimics) are used. In contrast, to achieve inhibition of oncogenic miRNAs, single-stranded antisense RNA were used (antagomiRs) [164]. Du et al. performed functional analysis of *miR-210-3p* using miRNA mimics and found that *miR-210-3p* promoted aerobic glycolysis by regulating glycolytic genes downstream of *HIF-1α* and *p53*. This activity conferred a growth advantage to TNBC and anti-apoptotic activity, suggesting that *miR-210-3p* may be a valuable target for the treatment of TNBC [165]. In the case of gene silencing for tsRNAs, a similar approach as for miRNAs is utilized. Zhu et al. constructed a *tRF*^*Lys−CTT−010*^ knockdown model by small RNA inhibitors, which resulted in decreased G6PC protein levels and a significant reduction in cellular lactate production. Knockdown of *tRF*^*Lys−CTT−010*^ inhibited the proliferation, migration and invasion of TNBC cells in vitro [80]. Therefore, targeting *tRF*^*Lys−CTT−010*^, a regulator of G6PC, presents a promising approach for TNBC treatment.

RNA interference (RNAi) technology may be an effective method for treating breast cancer based on lncRNA and circRNA. In this strategy, exogenous or mimic double-stranded RNAs, such as short interfering RNAs (siRNAs) and shRNAs, are often used to specifically knock down target genes. For instance, Qin et al. demonstrated that silencing *lnc030* expression through shRNA transfection led to a significant reduction in SQLE expression, resulting in decreased cellular cholesterol synthesis and inhibition of BCSC stemness maintenance. Animal experiments further validated the effectiveness of *lnc030* or SQLE knockdown in reducing tumor-initiating ability and inhibiting tumor growth in vivo [112]. This highlights the potential of targeting *Lnc030* and its downstream signaling as an effective therapeutic option. Wang et al. used shRNA knockdown of *circSEPT9*, which led to the inhibition of glutamine uptake and cell proliferation in BC cells. Subsequent mouse model experiments corroborated the in vitro anticancer activity of circSEPT9 silencing [119]. In addition, as described previously, LncRNAs *HOXC-AS3 *[57], *DIO3OS *[72], *circKIF4A* [68], and *circZFR *[97] can regulate metabolism by targeting PFK-1, LHDA, PKM2, and FABP, which in turn exert pro-cancer effects. Silencing these ncRNAs may be a target for potential therapeutic effects in breast cancer.

In conclusion, the combination of targeting ncRNAs and metabolic therapy presents a promising therapeutic strategy that may offer new insights and solutions for individualized tumor treatment. However, further basic research and clinical practice are necessary to validate its safety and efficacy.

#### Biomarkers

Early detection, diagnosis and treatment are key to improving the prognosis of breast cancer. Currently, there are still limited methods for predicting postoperative outcomes as well as testing for treatment efficacy. Therefore, the search for accurate biomarkers is crucial for early diagnosis and accurate prognosis of breast cancer. Numerous findings have shown that dysregulated ncRNAs expression is observed in breast cancer and ncRNAs are expected to serve as a diagnostic and prognostic biomarker.

There is growing evidence that metabolism-regulating ncRNAs play an important role as biomarkers in the diagnosis of BC, by being detected in breast cancer tissue or fluids. As mentioned previously, Li et al. found that *LncRNA PlncRNA-1* inhibited breast cancer growth by down-regulating PHGDH, the enzyme that catalyzes the first step of the serine biosynthetic pathway. The authors used ROC curve analysis to evaluate the diagnostic value of PlncRNA-1 expression in breast tissue and serum for breast cancer. The area under the curve (AUC) of *PlncRNA-1* expression in breast tissue in the diagnosis of breast cancer was 0.8994, and the AUC of serum *PlncRNA-1* in the diagnosis of breast cancer was 0.8667. suggesting that PlncRNA-1 can accurately predict breast cancer in both tissue and serum [133]. Huang identified differentially expressed tRFs in normal and breast cancer cell lines, and the AUCs of *tDR-7816, -5334,* and -*4733* were 0.859, 0.661, and 0.621, respectively, according to the ROC curve results. It can be inferred that *tDR-7816, tDR-5334* and *tDR-4733* may serve as potential candidates for non-TNBC breast cancer biomarkers. Functional analysis of target genes showed that the target genes of these three tRFs play a role in lipid metabolism processes such as glucuronic acid metabolism, steroid metabolism, and lipid biosynthesis [107]. Therefore, ncRNAs that regulate metabolism could serve as potential diagnostic markers for breast cancer.

**Fig. 5:**
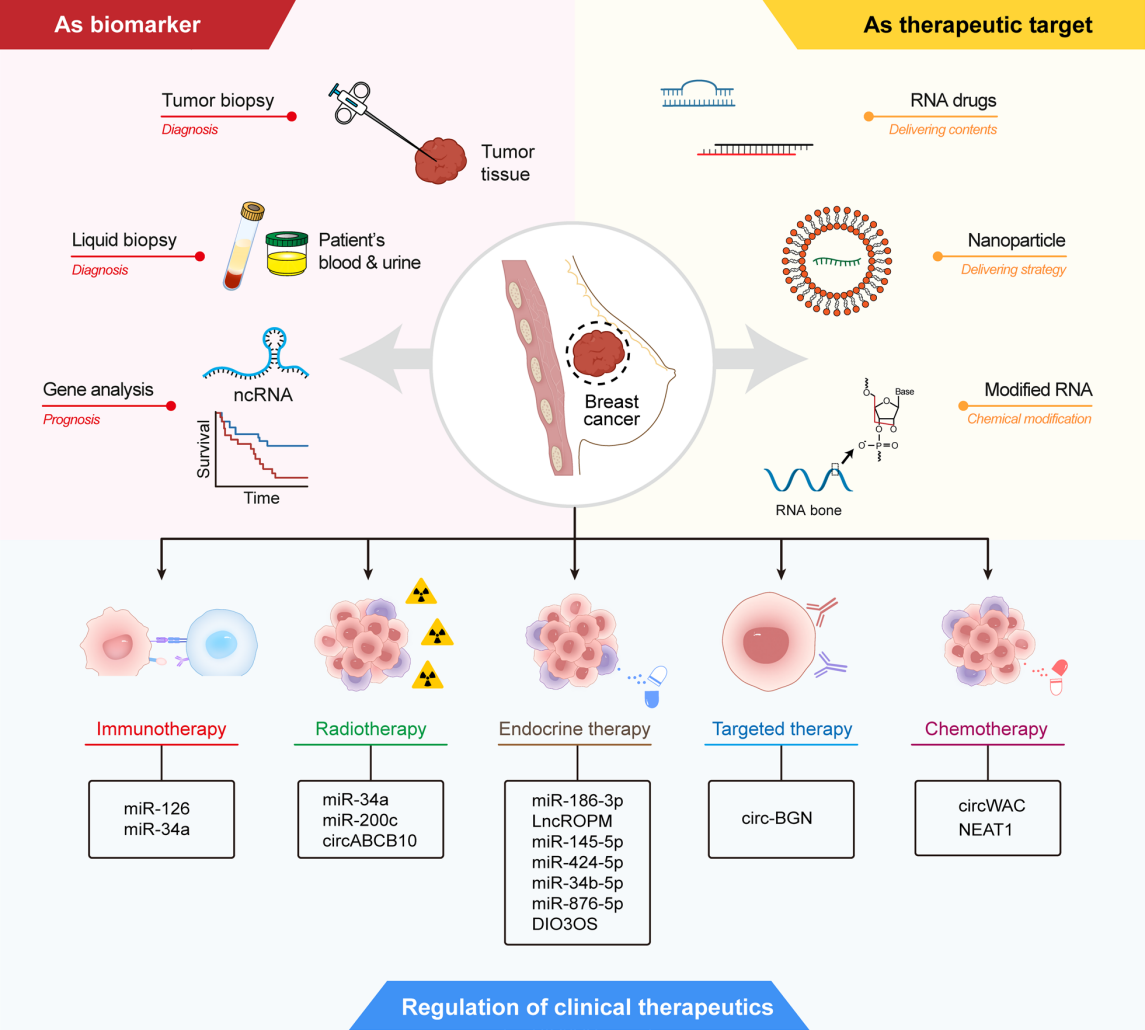
Clinical implications of non-coding RNAs and metabolism in breast cancer

Determining the prognostic value of metabolism-regulating ncRNAs is an essential field of BC research. The lncRNA breast cancer anti-estrogen resistance 4 (BCAR4) is required for YAP-dependent glycolysis. The expression levels of *BCAR4* and YAP were positively correlated in tissue samples from breast cancer patients, where high expression of *BCAR4* and YAP was associated with poor survival prognosis [90]. *CircPDCD11* accelerated the rate of glucose uptake, lactate production and extracellular acidification in TNBC cells. Clinical results showed that high *circPDCD11* expression was closely associated with poor prognosis and was an independent risk factor for TNBC prognosis [73]. *circRNF20* can promote breast cancer cell proliferation and aerobic glycolysis through the *circRNF20*/*miR-487a*/HIF-1α/HK2 axis. The expression of *circRNF20* was closely associated with lymph node metastasis and tumor size. Thereby, upregulation of *circRNF20* expression indicated a poor prognosis [89]. *miR-16-13p* inhibited aerobic glycolysis by suppressing PGK1. *miR-16–1-3p* expression was negatively correlated with breast cancer lung metastasis and negatively correlated with tumor size, lymph node status and grade, suggestive of a good prognosis [70]. *miR-128* inhibited glucose metabolism, mitochondrial respiration and proliferation in TNBC cells. Low expression of *miR-128* was associated with shorter overall survival and disease-free survival in TNBC, with shorter overall survival in non-TNBC, and not with disease-free survival [166]. Therefore, there is growing evidence that ncRNAs regulating metabolism can be applied not only as diagnostic biomarkers but also as prognosticators for breast cancer (Fig. 5).

## Discussion

Metabolism plays a central role in maintaining cellular homeostasis and responding to internal and external stimuli. Tumor cells often undergo metabolic reprogramming to support their rapid proliferation and anabolic reactions. Non-coding RNAs can maintain the balance of cellular metabolism by regulating genomic function, or they can establish cancer cell-specific metabolic networks that contribute to cancer development. This review explores how ncRNAs influence metabolic processes in breast cancer, including: (1) influencing transporter proteins on cell membranes to regulate nutrient transport, (2) modulating key enzymes in metabolic pathways to regulate energy flow, (3) influencing the ability of breast cancer cells to adapt and thrive under conditions of nutrient deficiency. Metabolic regulation and reprogramming mediated by ncRNAs ultimately may cause treatment resistance and poor clinical outcomes. Therefore, leveraging ncRNAs targeting metabolism in combination with existing therapeutic approaches can enhance treatment efficacy and improve patient prognosis. These ncRNAs can act as sensitizers, making tumors more susceptible to treatment. Moreover, targeting metabolism-regulating ncRNAs themselves can be a viable therapeutic strategy. Moreover, metabolism-regulating ncRNAs can be detected as a biomarker, which can facilitate clinical diagnosis, disease staging, and prognostic evaluation of breast cancer.

It should be noted that although the clinical significance of non-coding RNAs in breast cancer metabolism has been widely studied and discussed, there are still many outstanding issues to be resolved. Firstly, existing studies primarily focus on glucose, fatty acid, and amino acid metabolism. To achieve a comprehensive understanding of their role, studies should broaden their scope to include other pathways, such as nucleotide metabolism. Secondly, while many studies have explored the phenotypic effects of metabolism-regulating ncRNAs, a deeper understanding of the underlying molecular mechanisms is still necessary. In addition, there is competition for nutrients between tumor cells and immune cells in the TME. Altered metabolic patterns of tumor cells can affect the metabolism of other cells in the TME, resulting in immunosuppressive effects. The metabolic regulation of non-coding RNAs between breast cancer cells and cells in the TME still needs further investigation. Finally, while ncRNAs regulating metabolism are being explored as potential biomarkers, their detection is mainly limited to serum or tissue specimens. With the emergence of liquid biopsy as a clinical assay, these ncRNAs can be non-invasively detected in body fluids like urine and saliva. The sensitivity and specificity of the assay as biomarkers need to be improved. In addition to this, although there is promise in leveraging ncRNAs for metabolic regulation as a therapeutic approach, the translation of these findings into clinical applications is still in its early stages. The complex interactions between tumor cells and the tumor microenvironment are a major impediment to metabolic therapy. Metabolic therapies may suppress anti-tumor immunity while inhibiting tumor progression [162]. Future optimization of metabolic therapies should aim to promote their synergistic effects with anti-tumor immunity.

As a matter of fact, there is temporal and spatial heterogeneity in tumor metabolism. As tumors progress from precancerous lesions to locally invasive tumors to metastatic cancers, metabolic phenotypes and vulnerabilities evolve [167]. For breast cancer, different metabolic preferences exist for different molecular subtypes. Even for TNBC subtypes, they can be categorized into adipogenic subtypes, glycolytic subtypes, and mixed subtypes based on metabolic profiles [168–170]. Developing different therapeutic strategies for the metabolic vulnerabilities of breast cancer presents both opportunities and challenges. Therefore, more advanced methods for assessing metabolic phenotypes, such as metabolomics, metabolic imaging, single-cell, and spatial assays, are needed. Tailoring personalized therapeutic strategies by analyzing a patient's unique tumor metabolic profile serves as a viable future direction.

## Conclusion

This review summarizes the roles and mechanisms of non-coding RNAs that regulate metabolism in breast cancer. Non-coding RNAs that regulate metabolism can have an impact on resistance to existing treatment modalities in breast cancer. In addition to this, they can serve as therapeutic targets and biomarkers in their own right. Therefore, therapeutic approaches targeting non-coding RNAs and metabolism present both opportunities and challenges in the future treatment of breast cancer.

## Data Availability

Not applicable.

## Abbreviations

ncRNAs: Non-coding RNAs
TCA: Tricarboxylic acid
miRNA: MicroRNA
LncRNA: Long non-coding RNA
circRNA: Circular RNA
tsRNA: TRNA-derived small RNA
miRISC: MiRNA-induced silencing complex
ceRNAs: Competitive endogenous RNAs
tRFs: TRNA-related small RNA fragments
tiRNAs: TRNA-derived stress-induced RNAs
SLCs: Solute carriers
GLUT: Glucose transporter
ECAR: Extracellular acidification rate
EREG: Epidermal regulator
HK2: Hexokinase 2
PDK1: Pyruvate dehydrogenase kinase 1
PFK-1: Phosphofructokinase-1
PK: Pyruvate kinase
G6P: Glucose-6-phosphate
GAPDH: Glyceraldehyde-3-phosphate dehydrogenase
F6P: Fructose 6-phosphate
F-1,6-BP: Fructose 1,6-bisphosphate
F-2,6-BP: Fructose-2,6-bisphosphate
PFKFB: PFK-2/FBPase
PEP: Phosphoenolpyruvate
PKM2: Pyruvate kinase isoenzyme M2
PHD3: Prolyl hydroxylase 3
hnRNPF: Heterogeneous nuclear ribonucleoprotein F
PGK1: Phosphoglycerate kinase 1
OCR: Oxygen consumption rate
LDHA: Lactate dehydrogenase
PDH: Pyruvate dehydrogenase
PTBP1: Polypyrimidine tract binding protein 1
PDHX: Pyruvate dehydrogenase protein X
GS: Glycogen synthase
GP: Glycogen phosphorylase
PGM: Phosphate glucose metastases
EMT: Epithelial–mesenchymal transition
G6PC: Glucose-6-phosphatase catalytic
TNBC: Triple-negative breast cancer
FABPs: Fatty acid binding proteins
ACC: Acetyl CoA carboxylase
FASN: Fatty acid synthase
FAO: Fatty acid oxidation
CPT-1: Carnitine palmitoyltransferase I
NEAT1: Nuclear paraspeckle assembly transcript 1
HMG-CoA: 3-Hydroxy-3-methylglutaryl CoA
MVA: Mevalonate
HMGCR: HMG-CoA reductase
SQLE: Squalene epoxidase
MOS: 2,3(S)-monoxysqualene
PCBP2: Poly(rC)-binding protein 2
BCSCs: Breast cancer stem cells
FFAs: Free fatty acids
PC: Phosphatidylcholine
PG: Glycerophosphoglycerol
SLC1A5: Solute carrier family 1 neutral amino acid transporter member 5
GPX4: Glutathione peroxidase 4
GSH: Glutathione
VGLUT2: Vesicular glutamate transporter 2
XBP1SBM: XBP1s binding micropeptide
SSP: Serine synthetic pathway
PHGDH: Phosphoglycerate dehydrogenase
PSAT: Phosphoserine aminotransferase
PSPH: Phospho serine phosphatase
3-PPyr: 3-Phosphohydroxypyruvate
AIs: Aromatase inhibitors
shRNAs: Small hairpin RNA
CTLA-4: Cytotoxic T-lymphocyte-associated antigen-4
PD-1: Programmed cell death receptor-1
PD-L1: Programmed cell death ligand-1
TME: Tumor microenvironment
ROS: Reactive oxygen species
LNA: Locked nucleic acid
siRNAs: Short interfering RNAs
AUC: The area under the curve
